# Donohue syndrome in an Egyptian infant: a case report

**DOI:** 10.1515/crpm-2021-0087

**Published:** 2022-12-28

**Authors:** Kotb Abbass Metwalley, Hekma Saad Farghaly, Lamiaa Mahmood Maxi

**Affiliations:** Department of Pediatrics, Faculty of Medicine, Assiut University, Assiut, Egypt

**Keywords:** Donohue syndrome, hyperglycemia, hyperinsulinemia, hypoglycemia, insulin-like growth factor 1

## Abstract

**Objectives:**

We aim to report a case of Donohue syndrome (DS) which is a rare genetically encoded, autosomal inherited recessive disorder linked with severe insulin-resistant diabetes.

**Case presentation:**

We hereby report a case of a 4 month -old girl infant with DS. The patient exhibited dysmorphic facial features, severe growth retardation, fasting hypoglycemia, postprandial hyperglycemia, and hyperinsulinemia which are the hallmarks of DS. The diagnosis of DS was confirmed by genetic analysis. The patient was treated with high-dose insulin and frequent nasogastric formula milk feeding to achieve reasonable glycemic control.

**Conclusions:**

We reported a typical case of DS in a 4-month-old female infant characterized by peculiar dysmorphic features and failure to thrive. She also fulfilled the biochemical criteria of fasting hypoglycemia, postprandial hyperglycemia, and severe hyperinsulinemia. The diagnosis was confirmed by a molecular genetic study. Our patient achieved reasonable glycemic control after treatment with high-dose insulin.

## Introduction

Donohue syndrome (DS) is a rare [<1/1,000 000] genetically encoded, autosomal inherited recessive disorder connected with severe insulin-resistant diabetes [1]. Mutations in the insulin receptors gene which is located at 19p13.2 have been linked to its pathogenesis [2]. It was first described in 1948 by Donohue [3]. The diagnosis of DS depends on the combinations of typical dysmorphic features and laboratory findings (fasting hypoglycemia and postprandial hyperglycemia associated with high levels of C peptide and insulin levels) supported by genetic analysis which relies on the detection of IR gene mutation that is responsible for its occurrence [4]. Treatment of DS requires a multidisciplinary team, which include Dermatologist and Pediatric Endocrinologist. A limited therapeutic option is available so far for this syndrome. In addition to the use of high-dose insulin, the use of recombinant human insulin-like growth factor-1 (rhIGF-1) in the treatment may be clinically useful, effective and may result in an increased lifespan [5].

## Case presentation

**Figure 1:**
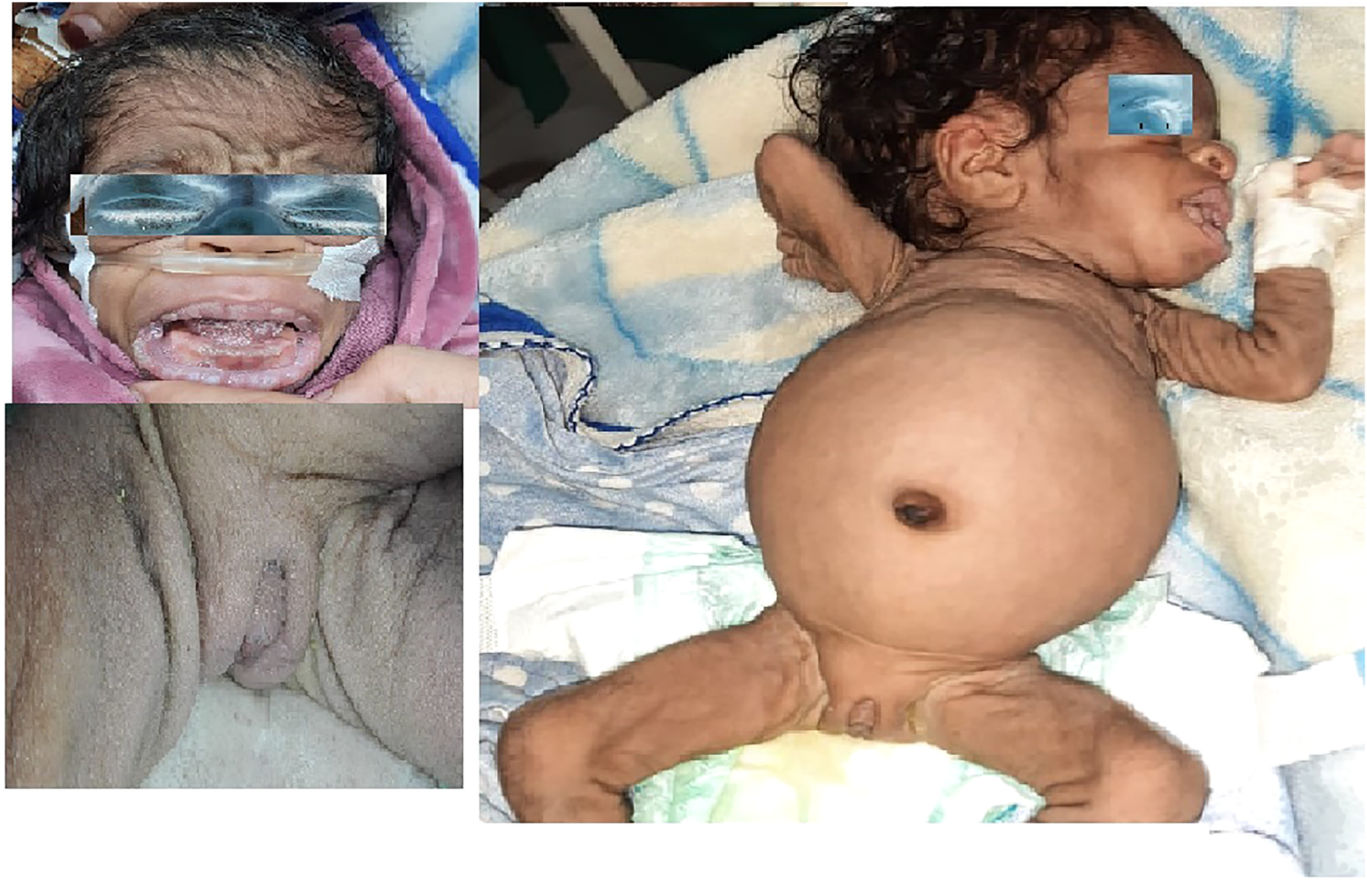
An infant with Donohue syndrome.

A 4-month-old girl was admitted to our institution for evaluation and management of poor growth. She was the fourth baby of consanguineous parents; one of her brothers died at the age of 2 months due to sudden infant death syndrome with the other brothers being normal. She was delivered by cesarean section at 38 weeks gestation due to intrauterine growth retardation. Her birth weight was 1240 *g*, her length was 37.3 cm and her head circumference was 30.5 cm. At the age of 12 days, she was admitted to the neonatal ward for suspected sepsis for a week and discharged in good general condition. On examination, she was found to be severely growth retarded with normal mentality, her weight was 2.5 kg, her length was 47 cm while the head circumference was 36 cm (all growth parameters below the 3rd centile). Clinical examination revealed abnormalities of the craniofacial region with elfin facies, protruding, deformed ears, big widely placed eyes (Figure 1), depressed nasal bridge, overgrown gum, prominent nipples, umbilical hernia, abdominal distension, dark skin (acanthosis nigricans), lipodystrophy, wrinkled hairy loose skin, and wrinkled loose skin and abnormal body proportions with very big feet and palm. Examination of the genitalia showed a big clitoris without palpable gonads (Figure 1). Cardiac auscultation revealed a murmur suggestive of ventricular septal defect (VSD) which was confirmed by echocardiography. The rest of the systemic examination was within normal limits. Investigations revealed hypoglycemia with fasting blood glucose ranging between 33 mg% to 46 mg%, and high levels of postprandial glucose ranging between 299 mg% and 476 mg% in repeated measurement, insulin level was high (345 uIU/ml, N: 2–25 uIU/ml) and C-peptide levels (22 ng/mL). FT4 level was 1.4 ng/dL (normal range:0.7 to 2.0), TSH level was 3.2 uIU/mL (normal range: 0.4 to 5.0), ACTH, cortisol, and 17 hydroxyprogesterone levels were normal. The assay of basal GH level was high (29.4 ng/mL), on the other hand, the level of IGF‐1 was low (30 μg/L), normal level (82–166 μg/L), and insulin-like growth factor-binding protein three was 0.15 mg/L (N: 0.81–1.9). MRI of the abdomen was normal. An echocardiographic study revealed a VSD with no evidence of cardiomyopathy. The chromosomal study was normal [46, XX]. A molecular genetics study revealed IR gene mutation responsible for the occurrence of DS. The parents were heterozygous carriers of this mutation. According to the above-mentioned data, a diagnosis of DS was made. Our patient accomplished control of blood glucose that was maintained between 78 and 146 mg/dl and glycated hemoglobin (HbA1c) of 7.8% on insulin glargine 1.6 unit/kg divided into two doses per day,with frequent nasogastric formula milk feeding. She was discharged from the hospital with the recommendation of follow-up in the outpatient clinic for repeating echocardiography and arranging the schedule for treatment by rhIGF-1.

## Discussion

The peculiar features of DS include a combination of a variety of physical features and metabolic symptoms, such as growth retardation hirsutism, acanthosis nigerians, lipoatrophy, clitoromegaly, fasting hypoglycemia postprandial hyperglycemia, insulin resistance, and hyperinsulinemia [4]. However, many of the criteria associated with DS differ in expression and severity from patient-to-another [6]. Differential diagnosis of the disorders that share mutations in the INSR gene, include type-A insulin resistance syndrome, Rabson Mendenhall syndrome,and DS. These disorders represent a continuum or spectrum of diseases. Patients with Rabson-Mendenhall syndrome have a moderate form in severity and may survive to adult age [7], while type-A insulin resistance syndrome affects pubertal girls and presented with hyperandrogenism, acanthosis nigricans, and hyperinsulinism [8].

Our case presented with marked hyperglycemia, profound hyperinsulinemia associated with hypoglycemia. This indicates defective insulin secretion and function at the same time. Hypoglycemia in DS may be attributed to an accelerated fasting state secondary to insulin resistance, scarce stores of glycogen, and deficient gluconeogenesis [9].

The typical cardiac affection in DS is hypertrophic cardiomyopathy which is attributed to severe hyperinsulinism [10]. However, our index case had VSD which is not reported before in DS, High consanguinity rate, genetic and environmental factors may be associated with certain congenital heart diseases such as ASD and VSD [11]. Therefore, echocardiography should be a routine investigation in every child with DS to detect cardiac anomalies. Furthermore, the enlarged clitoris in the index is similar to other children with DS, in spite that ambiguous genitalia is rare in children with DS [12]. The hormonal profile of the adrenal gland of our index case revealed an intact androgen and steroid biosynthetic pathway. Intrauterine exposure to high androgen levels may explain the genital ambiguity in our case.

Our patient achieved reasonable glycemic control after treatment with a high dose of insulin glargine. This is in agreement with Geffner et al. [13] who reported an intact *in vitro* action with high insulin dose in spite of the presence of insulin resistance among children with DS. This may explain the response of our case to the insulin in a high dose.

The presence of a high growth hormone level in an association of low IGF1 suggests that our index case had growth hormone resistance. We were unable to perform a classic growth hormone stimulation test that may give the actual status of growth hormone due to the young age of the case. This is in agreement with Psiachou et al. [14]. who reported the same result. It is postulated that GH resistance was a secondary effect caused by downregulation of GH receptor activity in the presence of high levels of insulin proximal to the cell membrane, with consequent limitation of IGF-I formation and consequently cellular growth [14]. Another study confirmed that GH resistance in an infant with DS improved with rhIGF-1 treatment by maintaining a normal growth rate [15]. Moreover, rhIGF-1 therapy may have long‐term improvements in glucose control and counteract the secondary effects of hyperinsulinemia [16].

## Conclusions

We herein reported a typical case of DS in a 4-month-old female infant characterized by peculiar dysmorphic features and failure to thrive. She also fulfilled the biochemical criteria of fasting hypoglycemia, postprandial hyperglycemia, and severe hyperinsulinemia. The diagnosis was confirmed by a molecular genetic study. Our patient achieved reasonable glycemic control after treatment with high-dose insulin.
